# MPI‐CDG from a hepatic perspective: Report of two Egyptian cases and review of literature

**DOI:** 10.1002/jmd2.12159

**Published:** 2020-09-07

**Authors:** Tawhida Y. Abdel Ghaffar, Bobby G. Ng, Solaf M. Elsayed, Suzan El Naghi, Sarah Helmy, Nermine Mohammed, Ahmed El Hennawy, Hudson H. Freeze

**Affiliations:** ^1^ Yassin Abdel Ghaffar Charity Centre for Liver Disease and Research Cairo Egypt; ^2^ Department of Paediatrics, Faculty of Medicine Ain Shams University Cairo Egypt; ^3^ Sanford Burnham Prebys Medical Discovery Institute. Human Genetics Program La Jolla California USA; ^4^ Department of Medical genetics, Faculty of Medicine Ain Shams University Cairo Egypt; ^5^ Department of Paediatrics National Hepatology & Tropical Medicine Research Institute Cairo Egypt; ^6^ Pathology Department Cairo University Cairo Egypt

**Keywords:** genetic variants, liver involvement, mannose, MPI‐CDG, portal hypertension

## Abstract

MPI‐CDG is a rare congenital disorder of glycosylation (CDG) which presents with hepato‐gastrointestinal symptoms and hypoglycemia. We report on hepatic evaluation of two pediatric patients who presented to us with gastrointestinal symptoms. Analysis of carbohydrate deficient transferrin (CDT) showed a Type 1 pattern and molecular analysis confirmed the diagnosis of MPI‐CDG. Oral mannose therapy was markedly effective in one patient but was only partially effective in the other who showed progressive portal hypertension.

**SYNOPSIS**

MPI‐CDG is one of the rare, but potentially treatable, causes of liver disease in children. Similarities to common childhood gastrointestinal diseases in tropical countries are a challenge for early diagnosis and management.

**Abbreviations**
AFP: Alfa feto‐protein
ALT: alanine aminotransferase
ANA: antinuclear antibody
ASMA: anti‐smooth muscle antibody
AST: aspartate aminotransferase
AT III: antithrombin III
aTTG: antitissue transglutaminase
CBC: complete blood count
CDG: congenital disorder of glycosylation
CDT: carbohydrate deficient transferrin
CHF: congenital hepatic fibrosis
CMV: cytomegalovirus
EBV: Epstein Barr‐virus
EMA: anti‐endomysial antibody
Hb: hemoglobin
kPa: kilopascal
LKM: liver kidney microsomal antibody
LL: lower limb
Man‐6‐P: mannose 6 phosphate
MCL: midclavicular line
PT: prothrombin time
PV: portal vein
PVD: portal vein diameter
PVV: portal vein velocity
TIEF: transferrin isoelectric focusing
ULN: upper limit of normal
US: ultrasound
WBC: white blood cells

## 1 INTRODUCTION

MPI‐CDG, a rare autosomal recessive congenital disease, is caused by a mutation in the *MPI* gene and a reduction of the enzymatic activity of phosphomannose isomerase.^1^ This enzyme normally catalyzes the interconversion of fructose‐6‐phosphate and Man‐6‐P, which can also be produced by direct phosphorylation of mannose. Both of these pathways can contribute to protein N‐glycosylation^2^ allowing mannose to supplement depleted pools of Man‐6‐P. MPI‐CDG is unique among CDGs as patients do not show neurologic involvement and oral mannose therapy effectively treats some of its manifestations, transforming it from a lethal into a treatable disease.^3^

Marques‐da‐Silva et al classified liver involvement in CDG into two main groups: one with predominant/isolated liver involvement and the other with merely associated liver disease. MPI‐CDG belongs to the former group.^4^

We hereby report on two young children who presented to us with liver disease. Upon their evaluation the diagnosis of MPI‐CDG was confirmed by the pattern of TIEF and molecular testing. Oral mannose therapy was provided to both but their response to therapy was variable.

To the best of our knowledge this is the first report on MPI‐CDG patients from Egypt as well as the first from the Middle East using oral mannose for therapy.

Case 1 CDG‐001 was referred to us in November 2014 at the age of 2.5 years because of hepatomegaly accidentally discovered during examination for repeated attacks of severe diarrhea that necessitated hospital admission and fluid therapy. The first of these attacks was at the age of 2 years. Stool analysis showed Ascaris lumbricoides and Giardia lamblia infections. Despite treatment, no improvement was noted. Cow milk products were eliminated from his diet resulting in partial improvement of his diarrhea. Investigations showed microcytic hypochromic anemia (Hb = 8.7 g/dL) and leukocytosis (WBC = 29 × 10^3^/μL). His serum creatinine, blood glucose, ALT and bilirubin levels were normal. He had slightly elevated AST (49 U/L) and low albumin (2.1 g/dL). He tested negative for both aTTG IgA and EMA IgA antibodies, making the diagnosis of celiac disease unlikely.

CDG‐001 was born to non‐consanguineous parents at full term. He had a normal birth weight and was breastfed. His motor and mental development was normal.

Upon examination, his height and weight were at the 5th and 10th centile, respectively. He looked weak and ill with mild LL edema and puffiness of the eyelids. His chest examination showed no abnormality nor did his cardiovascular examination. Abdominal examination revealed a very firm liver 6 cm below the costal margin in MCL and a palpable spleen 3 cm below the costal margin.

**FIGURE 1:**
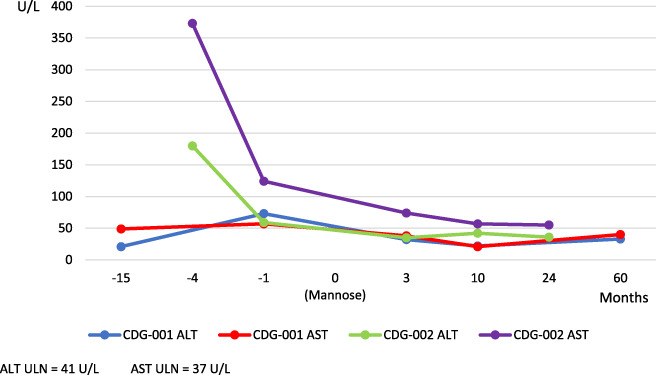
Liver enzymes pre and post mannose therapy. At presentation CDG‐001 had a mild elevation of both ALT and AST that persisted until the start of mannose therapy 15 months after presentation. Three months post mannose therapy they were both normal. They remained normal at 10 months post therapy and until last follow‐up. CDG‐002 had a moderate elevation of both ALT and AST (AST > ALT) at presentation. Although they were both substantially lower 3 months after start of mannose therapy, yet the normal values were not reached. Further drop occurred till 10 months post therapy. At last follow up, AST was still above normal

His ALT/AST fluctuated between being normal to slightly elevated (2‐3 times ULN) (Figure 1). Albumin was persistently low (3.3‐2.2 g\dl) but his PT was normal. CBC always showed high WBC (14.5‐18.5 × 10^3^/μL), low Hb (11.5‐12 g/dL) and normal platelet count. Albuminuria was absent. Viral markers (HBsAg, HBcAb, HCV antibody, CMV and EBV) and autoantibodies (ANA, ASMA & LKM) were negative. AT III activity was normal.

Fecal AAT was high (210 mg/dL, normal < 55 mg/dL), thus confirming protein losing enteropathy as the cause of hypoalbuminemia.

US/Doppler revealed hepatomegaly with homogenous echotexture and a patent PV (9 mm) with a hepatopetal flow (PVV 23 cm/s). Hepatic veins showed normal triphasic flow pattern. Fibroscan showed markedly increased stiffness (49 kPa).

Upper endoscopy and colonoscopy were done. Biopsies taken from the duodenum and the colon revealed mild non‐specific duodenitis and mixed focal colitis.

**FIGURE 2:**
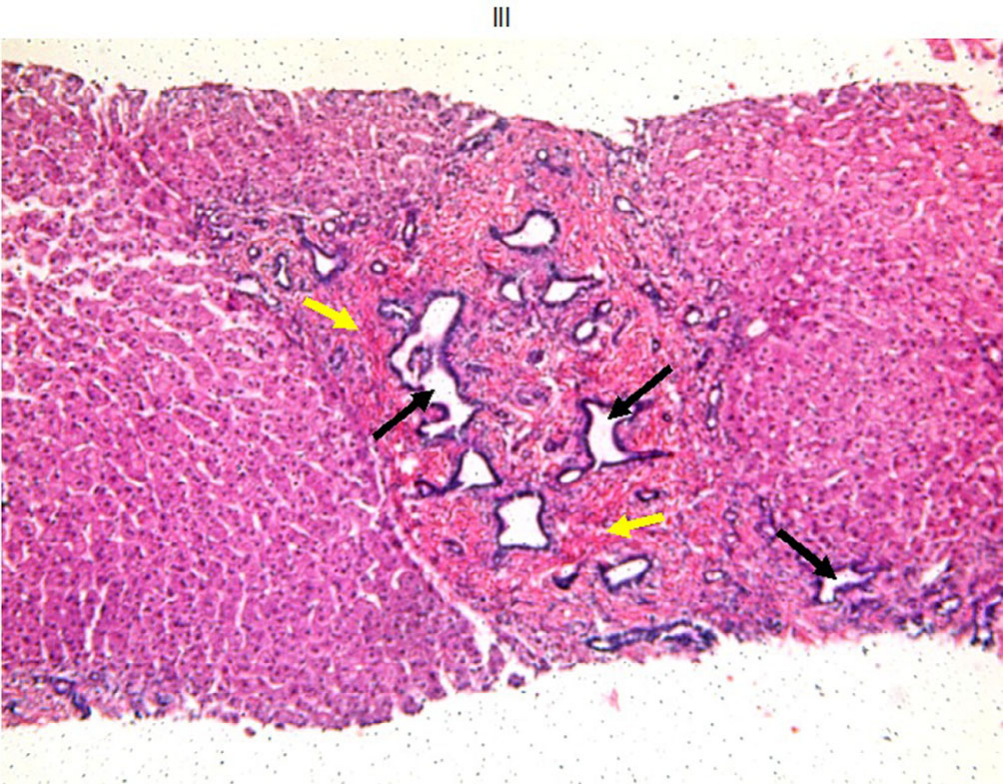
Liver biopsy of CDG‐001 showing portal tract expansions (yellow arrows) including aggregates of distorted dilated bile ducts (black arrows) consistent with a picture of ductal plate malformation which is a developmental anomaly that results from lack of ductal plate remodeling during bile duct morphogenesis. Inflammatory cells are absent. (H&E stain; original magnification 200×)

Liver biopsy showed the picture of CHF where broad fibrous septa including dilated distorted bile ducts were seen separating parenchymal islets (Figure 2).

We made a provisional diagnosis of MPI‐CDG based on the presence of firm hepatomegaly, splenomegaly, persistent hypoalbuminemia due to protein losing enteropathy, a histological diagnosis of CHF and absence of neurological symptoms. TIEF revealed elevation of disialo‐ and asialo‐transferrin, consistent with this diagnosis. Molecular testing showed compound heterozygosity for a novel c.487A>T (p.K163X) and a known c.656G>A (p.R219Q) pathogenic variant in *MPI* gene.

The child started mannose therapy at the age of 45 months (15 months after initial presentation to us) at a dose of 0.5 g/kg/day divided into five doses. This dose was gradually increased to 1 g/kg/day; since then his albumin has been always normal (4.7 g/dL). WBCs and Hb normalized (WBC 8 × 10^3^/μL, Hb 13 g/dL). His appetite improved. LL edema and eye puffiness disappeared, and the diarrhea became very infrequent. On his last examination at the age of 7.5 years his weight and height were on 50th centile. The liver was still palpable, but much less firm. His spleen was no longer palpable. Liver enzymes remained normal as did his CBC. Fibroscan done 1 year after the start of oral mannose showed a marked improvement in stiffness (13.7 kPa) although it was still in the F4 fibrosis stage.

Three years after initiation of mannose therapy, CDT test was 2.8% (slightly above the 2% cut‐off point) indicating near normalization of transferrin glycosylation.

Case 2 CDG‐002 was referred to us at the age of 14 months because of hepatosplenomegaly, ascites and elevated liver enzymes associated with decreased platelet count, low Hb and hypoglycemia which were noted while being investigated for repeated fever attacks and recurrent diarrhea.

CDG‐002 was born to non‐consanguineous parents with normal birth weight (3.5 kg). He was breastfed and had normal mental and physical development.

Upon examination, his height and weight were at 25th and 50th centiles, respectively and he looked ill and irritable. He had neither jaundice nor LL edema, but he had ascites. His liver was felt 3 cm below the costal margin in MCL, while his spleen was felt by ballottement. Abdominal wall veins were dilated and tortuous.

Blood tests revealed microcytic hypochromic anemia (Hb 9.5 g/dL), leukocytosis (WBC 14.2 × 10^3^/μL) and normal platelet count.

Both ALT and AST were high (120, 285 U/L respectively) (Figure 1), Albumin was 2.7 g/dL. GGT and bilirubin were normal. Prothrombin activity was 77% and ATIII activity was 56%. Random blood glucose was 53 mg/dL. Albuminuria was absent. Fecal AAT was high (150 mg%) confirming presence of protein losing enteropathy.

US/Doppler examination showed hepatosplenomegaly and ascites. The liver had a coarse echo pattern. A small single solid hypoechoic lesion (8 × 8.5 mm) was seen in the left lobe. PV was patent (7 mm) with hepatopetal flow (PVV 14 mm/sec). AFP was 32 ng/mL. Fibroscan showed an F4 stage of fibrosis (stiffness = 17.1 kPa).

**FIGURE 3:**
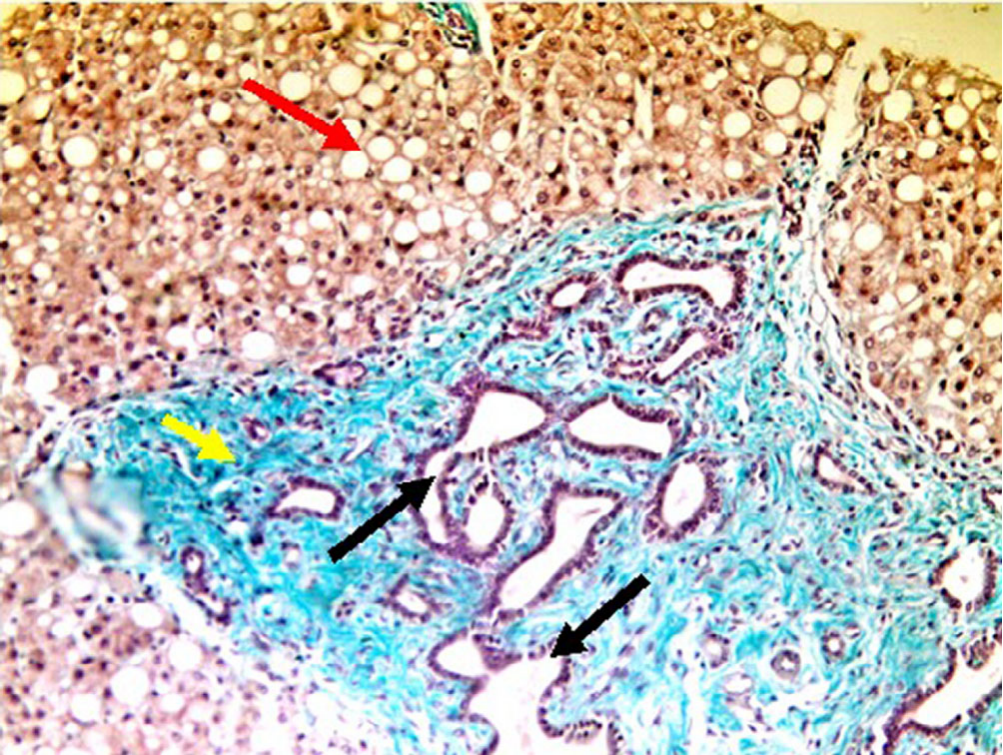
Liver biopsy of CDG‐002 showing extensive macrovesicular steatosis (red arrow) in addition to the same histological features as in Figure 1 (distorted dilated bile ducts (black arrows) within an expanded fibrous septum (yellow arrows)). This fat accumulation within hepatocytes underscores the severity of malnutrition and hypoglycemia that CDG‐002 has been suffering from. (Gomori trichrome stain; original magnification 200×)

Liver biopsy showed broad fibrous septa including dilated, distorted bile ducts and marked macrovesicular steatosis involving more than 50% of hepatocytes (Figure 3).

MPI‐CDG was suspected based on this constellation of manifestations. Type I TIEF pattern and presence of two previously reported *MPI* pathological variants: c.884G>A (p.R295H) and c.1193T>C (p.I398T) confirmed the diagnosis.

Mannose therapy was initiated 4 months after presentation at a dose of 0.2 g/kg/day divided into 4 doses. His diarrhea was reduced, and ascites resolved. He also developed a better appetite. His liver enzymes dropped. Hb increased, and WBC and blood glucose normalized. However, severe ascites recurred shortly thereafter. US/Doppler examination was repeated to exclude thrombosis of portal or hepatic veins. It showed ascites, an enlarged coarse liver with irregular surface, an enlarging spleen (11 cm) with PVD of 6 mm and PVV of 11 cm/sec.

Mannose dose was increased very gradually to 0.8 g/kg/day as he occasionally showed intolerance in the form of abdominal pain and mild diarrhea. Marked improvement in serum albumin level occurred. Ascites resolved. Currently he still cycles on and off ascites.

CDG‐002 developed melena at the age of 26 months. Endoscopic examination revealed grade III esophageal varices with signs of recent bleeding and a large gastric extension. As hemorrhoids were noted, colonoscopy was performed at the age of 30 months. It showed extensive rectal varices. Injection sclerotherapy was repeatedly done. Non‐selective beta‐blocker had to be prescribed for secondary prevention of variceal bleeding. CDT done 1 year after starting mannose was 10.9% (ref. < 1.7%). The mannose dose was recently increased to 1 g/kg/day divided into five doses.

## 2 DISCUSSION

The enzymatic and molecular basis of MPI‐CDG was described by.^5^ So far, only 35 patients with MPI‐CDG have been described in the literature.^6^ We herein add another two patients in whom clinical, laboratory, pathological and molecular features confirmed the diagnosis of MPI‐CDG.

Being a rare disease, MPI‐CDG diagnosis could be easily missed. This is especially true in developing countries where recurrent diarrhea, a nearly universal first manifestation of MPI‐CDG, is usually attributed to bacterial and parasitic causes (as in CDG‐001). Celiac disease and cow milk allergy are other increasingly diagnosed causes of diarrhea in infancy. Diarrhea was attributed to cow milk allergy and diagnosis was delayed in our first patient.

Hypoalbuminemia, another feature of MPI‐CDG, may as well be present in other disorders: malnutrition, hepatic, renal or intestinal disease. In both of our patients, protein losing enteropathy was evidenced by high fecal AAT. Malnutrition could be a contributory factor. Since many intestinal enzymes and proteins require normal glycosylation for full activity,^7^ therefore in defective glycosylation malnutrition may occur.

Glycosylation is the most important post translational modification of proteins. It is considered a fundamental cellular process.^8^ The liver is a major site of glycosylation. It is thus anticipated that liver development, structure and function is affected by defective glycosylation which would result in the accumulation of abnormal product or loss of function.^2^

Although liver involvement has been reported in only 22% of patients with various CDG types,^4^ it is a feature of nearly all N‐linked CDGs and is almost a constant feature of MPI‐CDG. It may occasionally develop later after other symptoms.^9^

Liver involvement ranges from asymptomatic hepatomegaly to elevated liver enzymes, ductal plate malformation, steatosis, and fibrosis. Even progressive portal hypertension, cirrhosis and liver failure have been described in MPI‐CDG (Table 1). Jaundice is rarely present. Liver involvement also results in abnormal synthesis of various proteins.

**TABLE 1:** Liver involvement in MPI‐CDG as reported in the literature and in our 2 patients

Liver Involvement	Reference	CDG‐001	CDG‐002
Hepatomegaly	de Koning et al^10^; de Lonlay et al^11^ Westphal et al^12^; Hendriksz et al^13^; Kelly et al^14^; Penel‐Capelle et al^15^; Damen et al^16^; Mention et al^17^; Hernández et al^18^; Janssen et al^19^; Deeb and Al Amoodi^20^	Yes	Yes
Elevated hepatic transaminases	de Koning et al^10^; Babovic‐Vuksanovic et al^9^; Hendriksz et al^13^; Mention et al^17^; Hernández et al^18^; Deeb & Al Amoodi^20^	Yes (Mild)	Yes (Moderate)
Liver failure	de Koning et al^10^; de Lonlay et al^11^	No	No
Portal hypertension	Pedersen & Tygstrup^21^; van Diggelen et al^22^; de Lonlay et al^23^; Westphal et al^12^; Liem et al^24^; Janssen et al^19^	No	Yes
Gastrointestinal bleeding	Pedersen & Tygstrup^21^; Westphal et al^12^; Liem et al^24^; Janssen et al^19^	No	Yes
Recurrent cholangitis	de Lonlay et al^11^	No	No
Fibrosis	de Koning et al^10^; Jaeken et al^25^; Adamowicz et al^26^; de Lonlay et al^11^; Hendriksz et al^13^; Westphal et al^12^; Kelly et al^14^; Damen et al^16^; Liem et al^24^; Mention et al^17^; Janssen et al^19^	Yes	Yes
Cirrhosis	de Lonlay et al^11^; Liem et al^24^	No	No
Steatosis	de Lonlay et al^11^; Kelly et al^14^; Zentilin Boyer et al^27^; Liem et al.,^24^	No	Yes
Ductal plate malformation/CHF/Von Meyenburg Complexes	Pedersen & Tygstrup^21^; de Koning et al^10^; Jaeken et al^25^; Babovic‐Vuksanovic et al^9^; de Koning et al^32^; Kelly et al^14^; Hendriksz et al^13^; Damen et al^16^; Mention et al^17^	Yes	Yes
Liver transplantation	Janssen et al^19^	No	Under consideration

Both of our patients presented with hepatosplenomegaly and elevated liver enzymes. Hepatomegaly has been reported as the most common clinical sign in MPI‐CDG. An enlarged spleen usually appears in the first year of life.^6^ CDG‐002 manifested signs of portal hypertension at presentation. Both children had ductal plate malformation and fibrosis in their liver. Prominent steatosis was additionally found in CDG‐002.

The course of liver disease and its response to therapy was different in CDG‐001 compared to CDG‐002. Throughout 44 months of follow up, the first child did not develop any new symptoms or signs of liver disease. His liver became less firm and his liver enzymes, INR and albumin normalized. On the other hand, the second child had a more severe hepatic phenotype. He presented at a younger age with portal hypertension, higher enzymes, and lower AT III. Despite near normalization of his enzymes and albumin, he had rapidly progressive increase in portal hypertension with development of melena and varices. Although oral mannose was started earlier in CDG‐002, yet the final dose was reached after a longer time due to intolerance.

Both patients were compound heterozygous for two different variants. CDG‐001 had a novel nonsense variant. His second variant, (c.656G>A; p.R219Q), is the most common pathogenic variant reported in 21.4% of the studied alleles.^6^ Interestingly, patients who are homozygous for this variant can be asymptomatic until adulthood.^28^ Those who are compound heterozygous for the c.656G>A (p.R219Q) and other variants may outgrow their symptoms without mannose therapy^12^ or have late presentation of diarrhea and excellent response to mannose therapy.^29^ The c.656G>A (p.R219Q) variant could be a disease‐ameliorating variant responsible for a milder phenotype in MPI‐CDG especially in regards to liver involvement.

CDG‐002 had two previously reported pathogenic variants. The c.884G>A (p.R295H) variant was first reported in a French‐Canadian patient. It was also reported in a heterozygous form in three parents of deceased children from the same region and so was suggested to be a founder mutation. Response to mannose therapy in these patients was not reported.^30^ Our patient did not have a French‐Canadian origin. The second variant c.1193T>C (p.1398T) was reported by de Lonely et al in a compound heterozygous form in a 3 months old child with typical MPI‐CDG and hyper insulinemic hypoglycemia who responded well to mannose therapy.^23^ It is suggested that phenotypes of *MPI* pathogenic variants are highly heterogeneous and likely modulated by various factors including the cellular import of mannose, the activity of phosphoglucose isomerase, diet variations affecting mannose availability and other unknown factors.^12^

Mannose therapy normalizes hypoglycemia, improves vomiting, diarrhea, the general clinical condition, and transferrin glycosylation.^2^ Yet mannose does not seem to correct the overall glycosylation profile^1^, ^12^ nor to substantially improve liver disease.^2^, ^17^ It does not prevent further hepatic injury. Liver improvement is probably dependent on the severity of the disease at diagnosis. Mannose may not be able to control a liver disease that is a consequence of a disorder of intrauterine development. In animal models, but not in humans, a link has been proposed between the progression of liver disease and the age at treatment initiation as well as the long‐term side effects of mannose.^31^

Oral Mannose therapy was well tolerated in CDG‐001. The dose was gradually and cautiously increased in CDG‐002 as rapid increases resulted in diarrhea. Recently, after reaching the full dose, CDG‐002 developed indirect hyperbilirubinemia due to hemolysis, a known side effect of mannose therapy. This was also seen in another MPI‐CDG patient during mannose therapy; she later had a liver transplant.^19^ Unfortunately, we could not measure mannose blood level in our patients as this test is not available in Egypt.

Liver transplantation is indicated in MPI‐CDG patients who develop progressive liver disease and its complications. Janssen et al^19^ reported the first successful liver transplantation for a 28‐years‐old patient with MPI‐CDG who developed hepato‐pulmonary syndrome. Liver transplantation is being considered for CDG‐002 because of his difficult to control, rapidly progressive portal hypertension.

From a hepatic perspective, screening for MPI‐CDG in patients with unexplained hepatomegaly, elevated liver enzymes or a picture of ductal plate malformation is worth undertaking especially if associated with other gastrointestinal symptoms or hypoglycemia. Because of the small number of reported cases it is difficult to draw conclusions neither on genotype‐phenotype correlation nor on genotype effect on response to oral mannose therapy. It is only through heightened awareness of this disorder that more cases may be diagnosed and studied.

## CONFLICT OF INTEREST

The authors declare no potential conflict of interest.

## ETHICS STATEMENT

All procedures followed were in accordance with the ethical standards of the responsible committee on human experimentation (institutional and national) and with the Helsinki Declaration of 1975, as revised in 2000.

## A PATIENT CONSENT STATEMENT

No patient identifiable information has been included in the manuscript. Oral consent was obtained from parents of both patients for inclusion of their case reports in this article.

## DOCUMENTATION OF APPROVAL FROM THE INSTITUTIONAL COMMITTEE FOR CARE AND USE OF LABORATORY ANIMALS

The manuscript does not contain any studies with human or animal subjects performed by any of the authors.
